# GALNT6 Promotes Tumorigenicity and Metastasis of Breast Cancer Cell via β-catenin/MUC1-C Signaling Pathway: Erratum

**DOI:** 10.7150/ijbs.67971

**Published:** 2021-11-11

**Authors:** Yingge Mao, Yuqi Zhang, Sairong Fan, Lvao Chen, Lili Tang, Xiaoming Chen, Jianxin Lyu

**Affiliations:** 1 Institute of Glycobiological Engineering, School of Laboratory Medicine and Life Sciences, Wenzhou Medical University, Wenzhou, Zhejiang, China; 2 Zhejiang Provincial Key Laboratory of Medical Genetics, Key Laboratory of Laboratory Medicine, Ministry of Education, China, School of Laboratory Medicine and Life Sciences, Wenzhou Medical University, Wenzhou, Zhejiang, China; 3 Hangzhou Medical College, Hangzhou, Zhejiang, China; 4 Present address: The First Affiliated Hospital of Henan University

In our paper 1, the image of invaded MDA-MB-231 cells with empty vector transfection (shRNA-NC) in Figure 3C was mis-pasted. The image of invaded MDA-MB-231 cells (Mock) was accidentally used for “shRNA-NC” in Figure 3C. Neither the interpretation nor the conclusion of this work is affected by this error. Figure 3C should be corrected as follows.

## Figures and Tables

**Figure 3:**
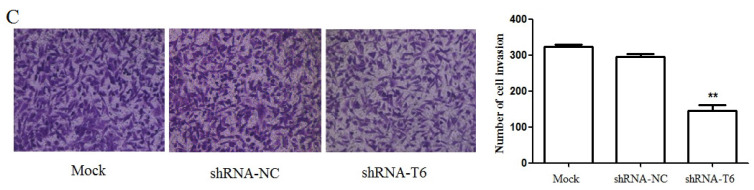
** Down-regulation of GALNT6 inhibited the migration and invasion capacity of MDA-MB-231 cells. (A)** Wound healing assay and quantification analysis showed that knockdown of GALNT6 significantly inhibited cell migration in MDA-MB-231cells. **(B)** Transwell migration assay and quantification analysis showed that knockdown of GALNT6 significantly inhibited cell migration in MDA-MB-231 cells. **(C)** Matrigel invasion assay and quantification analysis showed that knockdown of GALNT6 greatly inhibited invasive abilities of MDA-MB-231 cells. ** (D-E)** Nude mice (n = 5 per group) were injected with 1 × 10^7^ MDA-MB-231 cells (Mock, shRNA-NC or shRNA-T6) via the venous plexus of the eye. After 4 weeks, the mice were sacrificed under anesthesia. **(D)** The lungs were subjected to Bouin's fixation and photographed. A representative of the experiments is shown. Visible lung metastases nudes were counted. **(E)** The sections of lungs were stained with H&E. Data are expressed as means ± SEM. *P < 0.05 and **P < 0.01.
